# Identification of clinically relevant profiles in colorectal cancer through integrated analysis of bacterial DNA and metabolome in serum

**DOI:** 10.3389/fimmu.2025.1562416

**Published:** 2025-07-18

**Authors:** Juan Vicente-Valor, Sofía Tesolato, Dulcenombre Gómez-Garre, Mateo Paz-Cabezas, Adriana Ortega-Hernández, Constanza Fernández-Hernández, Sofía de la Serna, Inmaculada Domínguez-Serrano, Jana Dziakova, Daniel Rivera, Francisco-Javier Rupérez, Antonia García, Antonio Torres, Pilar Iniesta

**Affiliations:** ^1^ Department of Biochemistry and Molecular Biology, Faculty of Pharmacy, Complutense University, Madrid, Spain; ^2^ San Carlos Health Research Institute (IdISSC), San Carlos Hospital, Madrid, Spain; ^3^ Biomedical Research Networking Center in Cancer (CIBERONC), Carlos III Health Institute, Madrid, Spain; ^4^ Cardiovascular Risk Group and Microbiota Laboratory, San Carlos Hospital, Madrid, Spain; ^5^ Department of Physiology, Faculty of Medicine, Complutense University, Madrid, Spain; ^6^ Biomedical Research Networking Center in Cardiovascular Diseases (CIBERCV), Carlos III Health Institute, Madrid, Spain; ^7^ Centre for Metabolomics and Bioanalysis (CEMBIO), Faculty of Pharmacy, Universidad San Pablo CEU, CEU Universities, Madrid, Spain; ^8^ General and Digestive System Surgery Service, San Carlos Hospital, Madrid, Spain; ^9^ Department of Surgery, Faculty of Medicine, Complutense University, Madrid, Spain

**Keywords:** colorectal cancer, bacterial DNA, metabolome, diagnosis, serum biomarkers

## Abstract

**Introduction:**

There is increasing evidence demonstrating the relationship between microbiota and colorectal cancer. Several studies have been published analyzing microbiota in tissues and feces from cancer patients; however, there are only a few publications investigating the clinical utility of serum microbiome from colorectal cancer patients. Our aim was to advance in the search for serum biomarkers for the diagnosis of colorectal cancer.

**Methods:**

We conducted a cross-sectional study assessing bacterial DNA and metabolomic profiles in 64 serum samples from subjects affected by colorectal cancer and controls. A metagenomic analysis of the bacterial 16S rRNA gene in serum was established, and serum metabolites were detected through an untargeted metabolic study based on Gas Chromatography-Quadruple Time-Of-Flight Mass Spectrometry with accurate mass.

**Results and Discussion:**

After integrating the data resulting from the bioinformatics and statistical analyses, we obtained different profiles in colorectal cancer population and controls, regardless of the subjects' age, gender and body mass index. Serum levels of Firmicutes and threonic acid were the most relevant characteristics that could help differentiate both groups, achieving an excellent predictive accuracy in this discovery cohort (area under the ROC curve = 0.95). Although these results should be validated in other cohorts through multicenter studies, we consider that our data could be relevant and applicable to the early diagnosis of colorectal cancer.

## 1 Introduction

Colorectal cancer (CRC) is one of the neoplasms with the highest incidence and mortality worldwide. In 2022, 1.9 million people were diagnosed with CRC and more than 900,000 deaths resulted from this disease (1). CRC diagnosis requires the use of an invasive procedure such as colonoscopy (2), which is associated with a non-negligible rate of adverse events, mainly bleeding (<5%) and perforation (1:1000), at higher rates depending on patient characteristics such as age and comorbidities. Other cases (patients with a recent acute myocardial infarction or an acute intestinal inflammatory process) are contraindicated for this procedure (3). Non-invasive diagnostic tools such as colonic computerized tomography imaging or virtual colonoscopy and magnetic resonance imaging are performed in selected patients. However, molecular testing can potentially complement the current gold-standard techniques and improve accuracy (4). In this regard, the identification of biomarkers assisting the diagnosis of CRC from easily obtained samples, such as serum or stool, is highly requested. Hence, liquid biopsies have recently gained interest for cancer screening and diagnosis (5, 6).

Microbiota and metabolome have been identified as major contributors in carcinogenesis. Our commensal bacteria can exert pro-tumorigenic effects through immunological, toxicological and metabolic mechanisms (7, 8) which can be accomplished locally or distantly. Several “classic” locations serve as residence sites for microbiota species, including the gut, skin and respiratory and urinary organs. However, other non-traditional locations have emerged as potential carriers of microbial components, such as blood (9, 10) or serum/plasma (11, 12). Human serum is composed of the soluble, cell-free fraction of blood that remains after coagulation and centrifugation. Serum contains albumin, immunoglobulins, components of the complement system, electrolytes, and low or sequestered levels of key nutrients (13), but has also been described as carrier of bacteria or their structures, by means of nucleic acids detection, sequencing, imaging and culturing approaches (14). Previous investigations have further confirmed the presence of bacterial DNA in the blood from healthy subjects, its resemblance to the oral and gut microbiome, and its alteration regarding both microbiome load and composition in the presence of CRC or colorectal adenoma (15–17). The major fraction of the bacterial component of blood would come from the buffy coat, but plasma would also contain other minor components (18). However, the findings of a recent study, investigating blood bacterial DNA from a cohort of 9770 healthy subjects, support the hypothesis that its detection indicates a transient sporadic translocation of bacteria or their DNA from different body sites rather than the presence of a blood microbiome. Thus, the concept of a consistent and healthy blood microbial community is still under investigation, let alone the serum microbiome (19). The current knowledge about human blood microbiome and its potential as a prognostic marker for various diseases, such as cardiovascular disease, cirrhosis, pancreatitis, diabetes and chronic kidney disease, was explored in a recent review (20). Because the term “microbiome” has often been used inaccurately, in this study we specifically refer to bacterial DNA as the detection of bacterial 16S rRNA gene sequences through metataxonomic sequencing (21).

Similarly, metabolomics can be conceived as a comprehensive indicator of human health status (22). In cancer, a characteristic metabolic shift arises, reflecting the adaptation of tumoral tissues to the availability of nutrients from the environment to obtain energy for cell division. Therefore, some routes are upregulated, such as pentose phosphate and serine synthesis pathways, and others are deactivated, such as oxidative phosphorylation (Warburg effect) (23, 24). In addition, metabolites may be involved in cell proliferation, as they can trigger signaling processes and epigenetic regulation (25, 26).

Although serum microbial metagenomics, and specifically the one from whole blood, has been studied as biomarker for some diseases, the role of serum bacterial DNA as CRC biomarker remains unclear. We hypothesize that the identification of taxonomic microbiome signatures detectable in serum from patients with CRC, as well as their metabolic links, are useful in clinical oncology. The main aim of this study is the identification of serum biomarkers useful in the diagnosis of CRC.

## 2 Materials and methods

### 2.1 Patients and samples

For the study, a total of 64 serum samples from 43 CRC patients and 21 controls were collected and analyzed for bacterial DNA and metabolomic profiles. These samples were obtained prospectively between 2021 and 2024. All cases were recruited from the San Carlos Hospital in Madrid (Spain). Written approval to develop the study was granted from the Clinical Research Ethics Committee of the San Carlos Hospital (C.I. 19/549-E_BC, 27/12/2019). In addition, written informed consent was provided by all individuals prior to the investigation.

Regarding the CRC group, patients with no previous chemo- and/or radiotherapy undergoing curative-intention surgery were recruited subsequently and regardless of age, gender or tumor stage. Eligible controls were voluntary subjects without cancer or cancer history. The exclusion criteria in both groups were the presence of previous gastrointestinal resection surgery, inflammatory bowel diseases, or antibiotic treatment one month before surgery. **Table 1** shows the clinical-pathological variables of the study population. All individuals were categorized according to their body mass index (BMI) values, following the guidelines of the World Health Organization. CRC patients were staged following the American Joint Committee on Cancer classification (27).

**Table 1:** Clinico-pathological characteristics of the study population.

Variable	CRC group N=43	Control group N=21	*p* value (CRC *vs.* Control)
Age, Median in years (IQR)	76.00 (67.00-78.00)	57.00 (39.00-64.00)	< 0.001^$^
Gender, N (%)	43 (100.0)	21 (100.0)	0.027^#^
Male	27 (62.8)	7 (33.3)	
Female	16 (37.2)	14 (66.7)	
BMI group, N (%)	43 (100.0)	21 (100.0)	0.003^#^
Normal weight (BMI < 25 kg/m^2^)	10 (23.3)	3 (14.3)	
Overweight (BMI ≥ 25 kg/m^2^ and < 30 kg/m^2^)	21 (48.8)	3 (14.3)	
Obesity (BMI ≥ 30 kg/m^2^)	12 (27.9)	15 (71.4)	
Tumor location (CRC), N (%)	43 (100)		
Right colon	21 (48.8)		
Left colon	16 (37.2)		
Rectum	6 (14.0)		
TNM stage, N (%)	43 (100.0)		
I-II	21 (48.8)		
III-IV	22 (51.2)		

BMI, body mass index; CRC, colorectal cancer; IQR, interquartile range; TNM, tumor, node, metastasis; ^$^Mann-Whitney U test; ^#^Chi-squared test.

Blood was collected after an overnight fast, prior to the surgery in the case of CRC patients. Serum was separated and stored in aliquots at -80°C until processing.

### 2.2 DNA extraction and bacterial 16S rRNA gene sequencing

Total DNA was extracted from the serum samples using the QIAamp^®^ DNA Mini Kit (Qiagen, Hilden, Germany), following the manufacturer’s protocol for blood or body fluids, from a starting amount of 200 μL of serum and eluting DNA in a final volume of 50 μL of AE buffer. DNA was then quantified using the Invitrogen™ Qubit™ 3 Fluorometer with the dsDNA HS (high sensitivity) Assay (Thermo Fisher Scientific, Waltham, MA, USA). Two extraction blanks were also included, which underwent all extraction and sequencing steps along with the samples and were subsequently used for the correction of sample lectures.

Metagenomic analysis of serum bacterial DNA was performed via the amplification and sequencing of the bacterial 16S rRNA gene using the Ion Torrent™ sequencing technology and the reagents from Life Technologies S.A. (a part of Thermo Fisher Scientific), as described previously (28).

### 2.3 Serum untargeted metabolomics by gas chromatography-mass spectrometry

Metabolites contained in the serum samples from CRC patients and controls were subjected to an untargeted metabolomics approach by Gas Chromatography-Quadruple-Time-of-Flight Mass Spectrometry (GC-QTOF-MS), following previously published protocols (29, 30). Prior to analysis, 40 μL of each serum sample were combined with 120 μL of acetonitrile containing an internal standard and proteins were removed. The resulting extracts were subjected to a two-step derivatization procedure and the analysis of the final sample extracts was performed by an Agilent 7980B GC system coupled to an Agilent 7250 QTOF/MS analyzer (Agilent Technologies, Waldbronn, Germany).

### 2.4 Statistical and bioinformatic analyses

Metagenomic data were submitted to bioinformatic analysis using the Quantitative Insight Into Microbial Ecology 2 (QIIME2) pipeline (31). Raw Fastq files underwent a treatment that included pair-wise alignment and classification of reads with respect to the 12 possible forward and reverse primers, reorientation of the reverse reads and removal of primer sequences, and reads were all trimmed to 165 bp. The obtained sequences were submitted to further analysis for dereplication, singleton removal, assignment to operational taxonomic units (OTUs) and chimera filtering. Closed-reference OTU picking was performed using the SILVA 138 SSU Ref NR 99 identity database, based on a 99 % similarity between sequences. Mean count values from both blank OTUs were subtracted from the samples. Rarefaction was performed to a threshold over 6.5K OTUs. Alpha diversity was assessed on the rarefied OTU profiles through five metrics: observed OTUs, Chao1 richness estimate, Shannon diversity index, Pielou’s evenness index, and Simpson’s diversity index. Parametric tests (t-test) or non-parametric tests (Kruskal–Wallis test) were performed depending on the distribution of data. Beta diversity was also analyzed through the permutation-based multivariate analysis of variance (PERMANOVA), the analysis of similarities (ANOSIM), and the analysis of multivariate homogeneity of variances (PERMDISP) tests, for which Bray-Curtis and Jaccard similarity indexes were compared. Principal coordinate analysis (PCoA) was used to plot the distances between groups. Regarding taxonomic analysis, the linear discriminant analysis (LDA) effect size (LEfSe) analysis (32) was used to identify differentially abundant taxa between groups. Taxa with a logarithmic LDA score (log10) > 2 and P-value < 0.05 were considered as statistically significant.

Regarding metabolomics, results were subjected to deconvolution and annotation through MS library search using the software MassHunter Unknowns Analysis 10.0 (Agilent Technologies) against our CEMBIO in-house personal compound data library, along with Fiehn Library. After that, data alignment was performed by MassHunter Mass Profiler Professional 15.1 software (Agilent Technologies). Then, a library with the compounds annotated was built and signal integration of selected target ions for all compounds was performed by MassHunter Quantitative Analysis 10.0 for QTOF (Agilent Technologies). Peaks and artifacts present in the blanks were discarded in the samples. Data matrix was obtained after normalization by d-31 palmitic acid.

Further statistical analyses were conducted using STATA IC16.1 (Stata-Corp LLC, College Station, TX, USA) and IBM^®^ SPSS^®^ Statistics software package version 29 (IBM Inc., Armonk, NY, USA). Differences in metabolite abundance (normalized peak area, NPA) were determined using the corresponding parametric (Student’s t test) or non-parametric (Mann-Whitney *U* test) tests depending on the variable distribution in the study groups. Next, logistic regression was performed to assess the predictive capability of the differential metabolites (NPA) and bacterial taxa (relative abundance, RA) after adjusting for age, gender and BMI. In all cases, P-value < 0.05 was considered for statistical significance. The diagnostic capability for CRC of the individual bacterial taxa and metabolites in serum samples was further assessed by the Receiver Operating Characteristic (ROC) curves, from which values of the area under the curve (AUC), the sensitivity and the specificity were calculated. The degree of discrimination of the ROC curves was considered as “acceptable” for AUC values between 0.7 and 0.8, “excellent” for values between 0.8 and 0.9, and “outstanding” for values above 0.9, following previous criteria (33). To obtain the curves, the abundance values of each bacterial taxon or metabolite were processed by the Cutoff Finder application (34), which allows to calculate the optimal threshold to distinguish CRC patients from controls. The threshold calculation followed the Euclidean distance method to optimize both sensitivity and specificity. Finally, a predictive panel for CRC was obtained by integrating both metagenomic and metabolomic data into a logistic regression model. The predictive capability of the panel was tested through the CRC probability (P(CRC)) values obtained in the logistic regression equation:


$$P\left(CRC\right)=\;\frac{1}{1+e^{\left(-C-\beta_{1}X_{1}-\beta_{2}X_{2}-\beta_{3}X_{3}\ldots -\beta_{k}X_{k}\right)}}$$

For a model containing k independent variables (X), C is the value of the constant and β the value of the regression coefficient for each variable. The values of P(CRC) for each sample were again processed by the Cutoff Finder application, and ROC curves were obtained following the Euclidean algorithm.

## 3 Results

### 3.1 Comparison of microbial diversity between serum samples from CRC patients and the Control group

**Figure 1:**
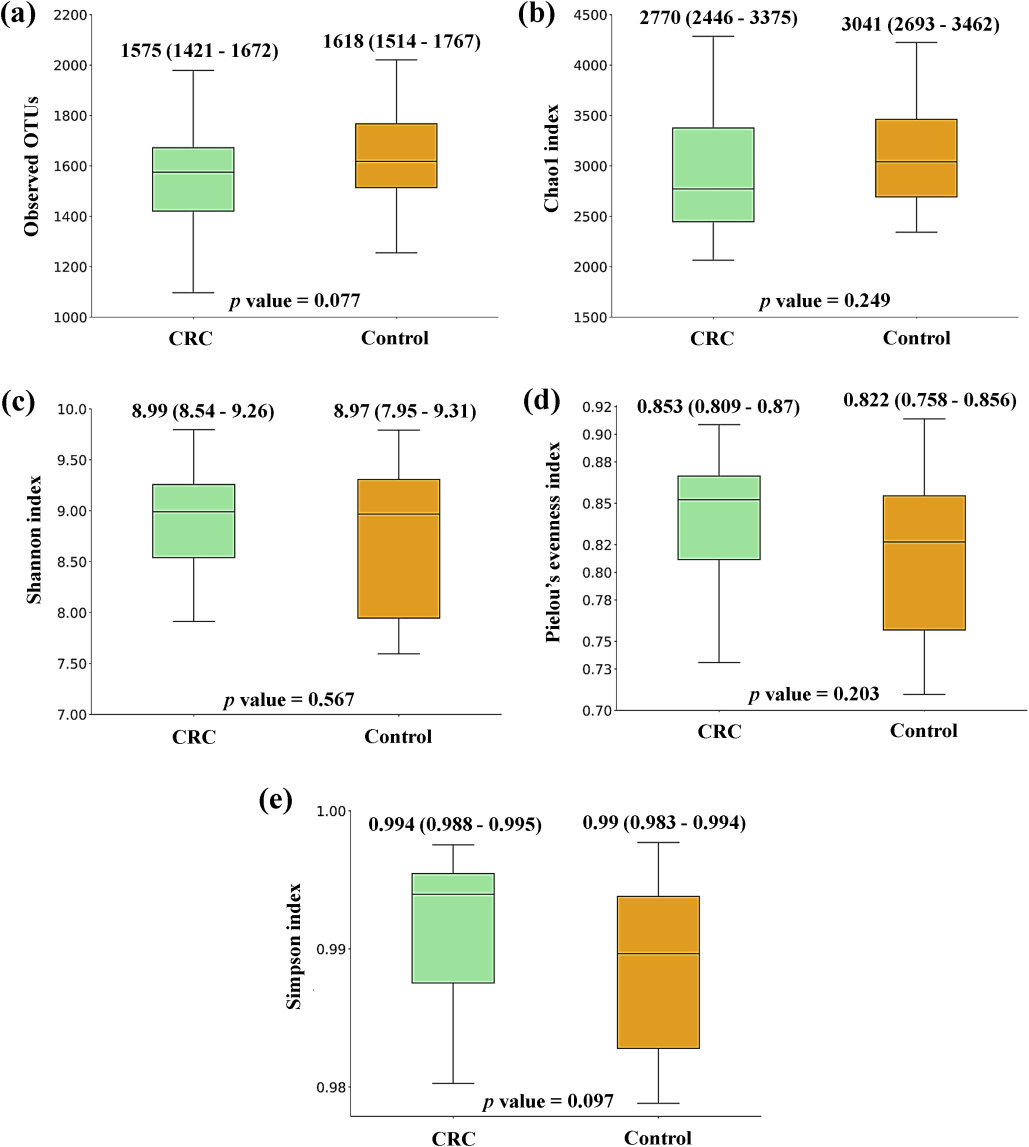
Alpha diversity comparison between serum samples from Colorectal Cancer (CRC) patients and controls. **(a)** Observed operational taxonomic units (OTUs) **(b)** Chao1 index **(c)** Shannon index **(d)** Pielou’s evenness index **(e)** Simpson index. Median values with interquartile range and P-values for Mann-Whitney *U* test are indicated. CRC n = 43 and controls n = 21.

**Figure 2:**
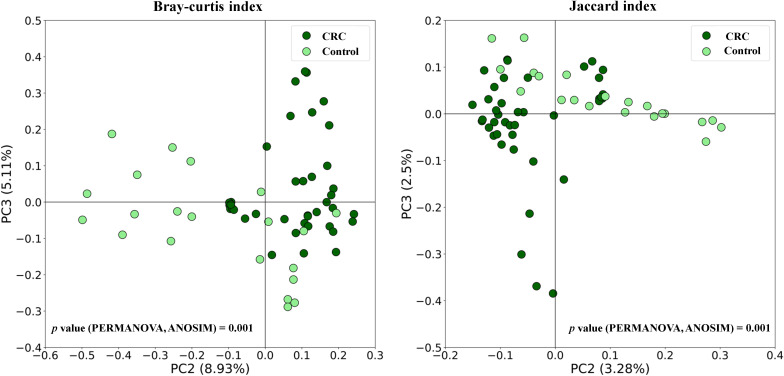
Beta diversity comparison between serum samples from Colorectal Cancer (CRC) patients and controls. Principal Coordinates Analysis (PCoA) plots based on Bray-Curtis and Jaccard indexes, and P-values for PERMANOVA and ANOSIM tests. CRC n = 43 and controls n = 21.

**Figure 1** shows the box plots and the P-value for the comparison of the five alpha diversity metrics (Observed OTUs, [a]; Chao1 index, [b]; Shannon index, [c]; Pielou’s evenness index, [d]; and Simpson index, [e]), performed between CRC and Control serum samples. As shown in the figure, no significant differences were found between both groups for none of the parameters. On the other hand, beta diversity was significantly different between both groups according to the PERMANOVA and the ANOSIM tests (in the two tests, P-value < 0.001 for both Bray-Curtis and Jaccard indexes). This could suggest that while the overall diversity within each sample (α-diversity) is similar, the composition and structure of those communities (β-diversity) is distinct. Bidimensional PCoA plots and P-value for both indexes are graphed in **Figure 2**, showing that both populations constitute distinguishable clusters.

### 3.2 Taxonomic comparison between CRC and control serum bacterial DNA

**Figure 3:**
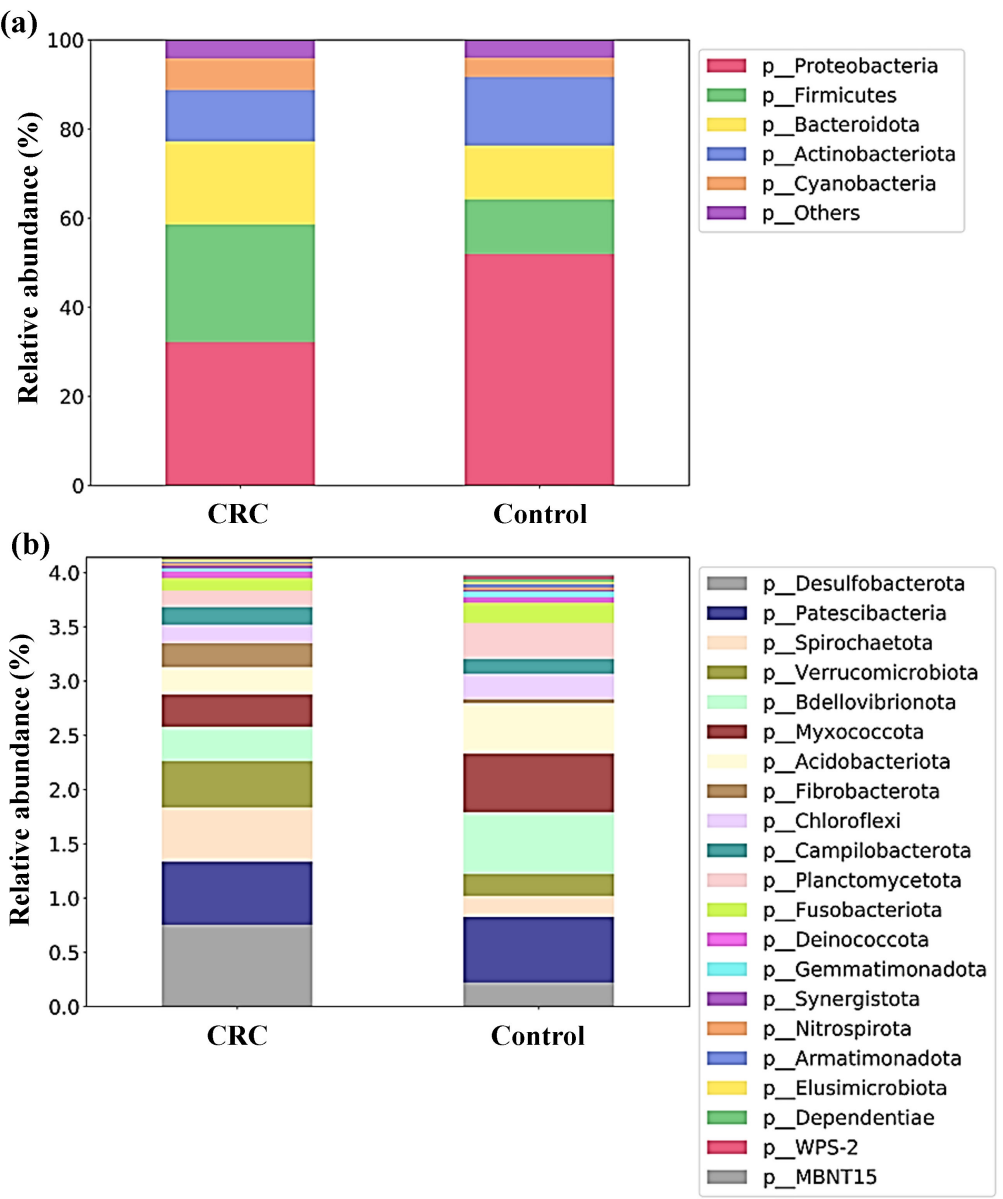
Proportion (relative abundances) of the bacterial phyla in serum samples from Colorectal Cancer (CRC) patients and controls. **(a)** Main bacterial phyla **(b)** Less abundant bacterial phyla. CRC n = 43 and controls n = 21.

When analyzing the taxonomic composition of serum bacterial DNA, both controls and CRC patients had marked differences reaching bacterial phyla. As shown in **Figure 3**, the average RA of the main bacterial phyla (grouped prevalence > 1%) (**Figure 3A**) was visibly different between both groups. Serum samples from controls had a clear preponderance of Proteobacteria and higher percentage of Actinobacteriota, whereas CRC serum samples had a decreased proportion of these phyla and increased presence of phyla Firmicutes and Bacteroidota.

Moreover, the proportion between Firmicutes and Bacteroidota, expressed through the Firmicutes to Bacteroidota (F/B) ratio of RA, also changed between both groups. In controls, both phyla showed similar proportions (F/B ratio of 1.01), whereas in CRC patients a preponderance of phylum Firmicutes was found (F/B ratio of 1.41).

**Figure 4:**
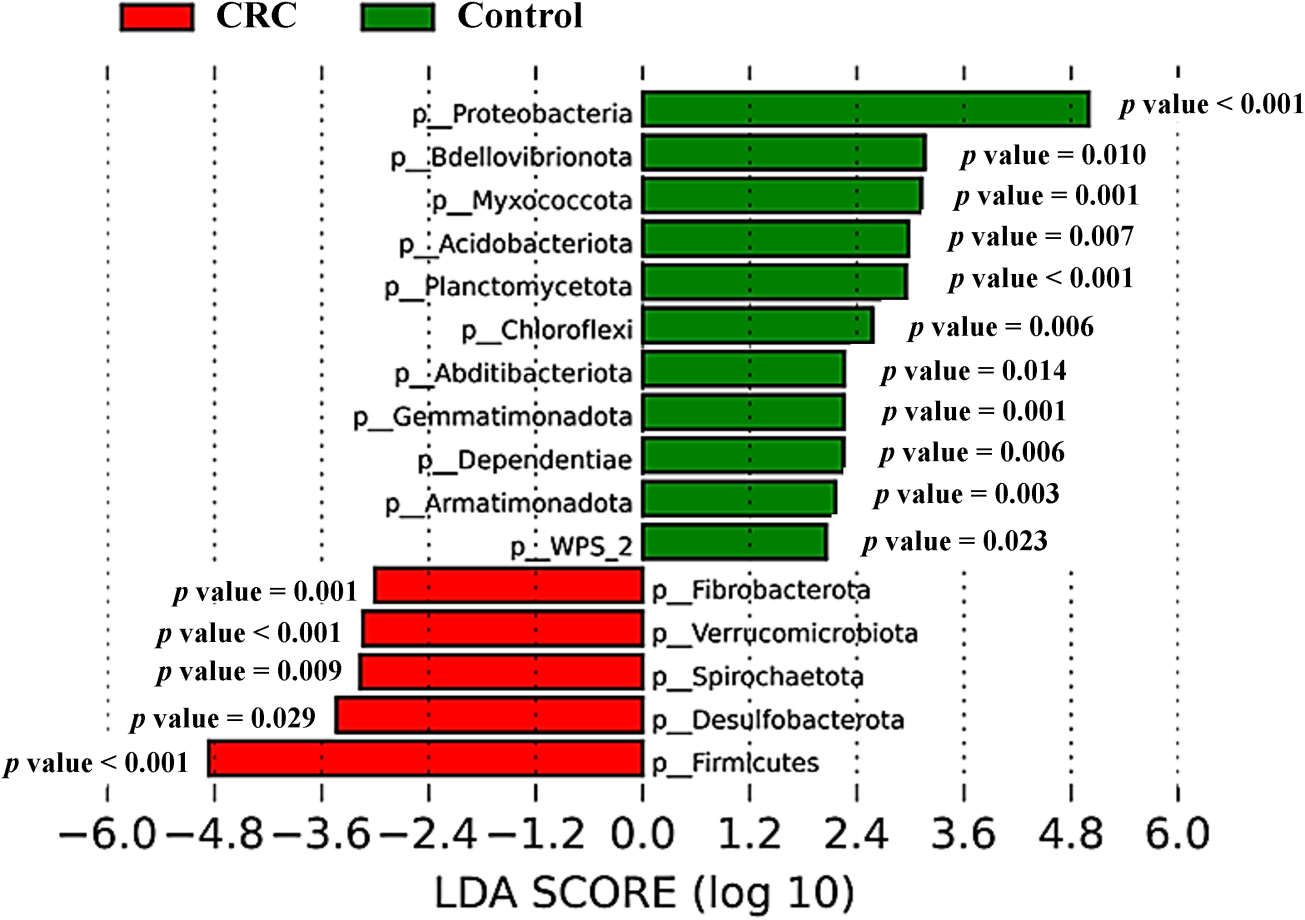
Taxonomic comparison between serum samples from Colorectal Cancer (CRC) patients and controls. Linear discriminant analysis Effect Size (LEfSe) analysis at the bacterial phylum level, with P-values for factorial Kruskal-Wallis test. CRC n = 43 and controls n = 21.

The different composition of both populations could also be noticeable for less abundant phyla (grouped prevalence < 1%) (**Figure 3B**), with some phyla (such as Desulfobacterota, Spirochaetota or Verrucomicrobiota) increased in CRC sera and others (like Bdellovibrionota, Myxococcota or Acidobacteriota) increased in control sera. Many of these observable differences were indeed reflected by the LEfSe analysis (**Figure 4**), which reported a significant increase in main phylum Proteobacteria, as well as in less abundant phyla Bdellovibrionota, Myxococcota, Acidobacteriota, Planctomycetota, Chloroflexi, Abditibacteriota, Gemmatimonadota, Dependentiae, Armatimonadota and WPS-2 in the serum from controls. On their counterpart, CRC sera had increased levels of the main bacterial phylum Firmicutes, and of less abundant phyla Desulfobacterota, Spirochaetota, Verrucomicrobiota and Fibrobacterota.

We found that 37 genera were increased in CRC after adjusting for confounders: *Bifidobacterium, Enterorhabdus, Bacteroides, Butyricimonas, Odoribacter, Muribaculaceae, Alloprevotella, Prevotella, Alistipes, Parabacteroides, Catenibacterium, Holdemanella, Turicibacter, RF39, Agathobacter, Anaerostipes, Blautia, Butyrivibrio, Coprococcus, Dorea, Lachnospira, Marbinbryantia, Roseburia, Butyricicoccus, Colidextribacter, Intestinimonas, Oscillibacter, CAG-352, Faecalibacterium, Ruminococcus, Phascholarctobacterium, Megamonas, Parasutterella, Esherichia-Shigella, Salmonella, Proteus, Akkermansia.* We continued to explore bacterial phyla instead of genera as biomarkers to develop a simpler diagnostic panel, considering the principle of parsimony, whereby if fewer variables can explain similar data, the use of fewer variables is encouraged.

Bacterial phyla that were either increased or decreased in CRC serum samples were tested for their predictive capability using logistic regression (**Tables 2**, **3**). After adjusting for confounders related to age, gender and BMI of individuals in a multivariate logistic regression, only Firmicutes and Verrucomicrobiota maintained a clear predictive effect. Regarding phyla that were decreased in CRC and increased in control serum samples (**Table 3**), seven of them (Proteobacteria, Bdellovibrionota, Myxococcota, Acidobacteriota, Planctomycetota, Armatimonadota and Gemmatimonadota) still had a clear predictive capability after adjusting for age, gender and BMI.

**Table 2:** Results from the logistic regression for the bacterial phyla increased in serum samples from Colorectal Cancer (CRC) patients with respect to controls.

Bacterial phylum	Unadjusted^*^ OR (95%CI)	Unadjusted^*^ *p* value	Adjusted^#^ OR (95%CI)	Adjusted^#^ *p* value
Firmicutes	1.189 (1.078-1.310)	< 0.001	1.190 (1.058-1.339)	0.004
Desulfobacterota	8.260 (1.244-54.828)	0.029	9.728 (0.938-100.942)	0.057
Spirochaetota	17.057 (0.593-490.400)	0.098	46.586 (0.401-5’414.529)	0.113
Verrucomicrobiota	127.715 (3.469-4’701.402)	0.008	98’302.064 (6.652-1.452E+9)	0.019
Fibrobacterota	325’700.324 (2.772-3.826E+10)	0.033	19’945.955 (0.868-4.583E+8)	0.053

CI, confidence interval; OR, Odds ratio; ^*^Univariate regression analysis; ^#^Multivariate regression analysis adjusting for age, gender and body mass index.

**Table 3:** Results from the logistic regression for the bacterial phyla increased in serum samples from controls with respect to colorectal cancer (CRC) patients.

Bacterial phylum	Unadjusted^*^ OR (95%CI)	Unadjusted^*^ *p* value	Adjusted^#^ OR (95%CI)	Adjusted^#^ *p* value
Proteobacteria	0.927 (0.888-0.968)	< 0.001	0.927 (0.880-0.975)	0.003
Bdellovibrionota	0.076 (0.010-0.560)	0.011	0.036 (0.003-0.406)	0.007
Myxococcota	0.031 (0.003-0.296)	0.003	0.026 (0.001-0.613)	0.024
Acidobacteriota	0.017 (0.001-0.307)	0.006	0.004 (0-0.163)	0.024
Planctomycetota	0 (0-0.037)	0.001	0 (0-0.126)	0.014
Chloroflexi	0.072 (0.002-2.207)	0.132	0.014 (0-1.446)	0.071
Abditibacteriota	0 (0-2’589.776)	0.144	3’835.830 (0-1.219E+27)	0.765
Gemmatimonadota	0 (0-3.972)	0.083	0 (0-0.142)	0.025
Dependentiae	0 (0-0.139)	0.033	0 (0-24’927.971)	0.181
Armatimonadota	0 (0-0.005)	0.016	0 (0-0)	0.009
WPS-2	0 (0-47.357)	0.152	0 (0-2.073)	0.061

CI, confidence interval; OR, Odds ratio; ^*^Univariate regression analysis; ^#^Multivariate regression analysis adjusting for age, gender and body mass index.

**Figure 5:**
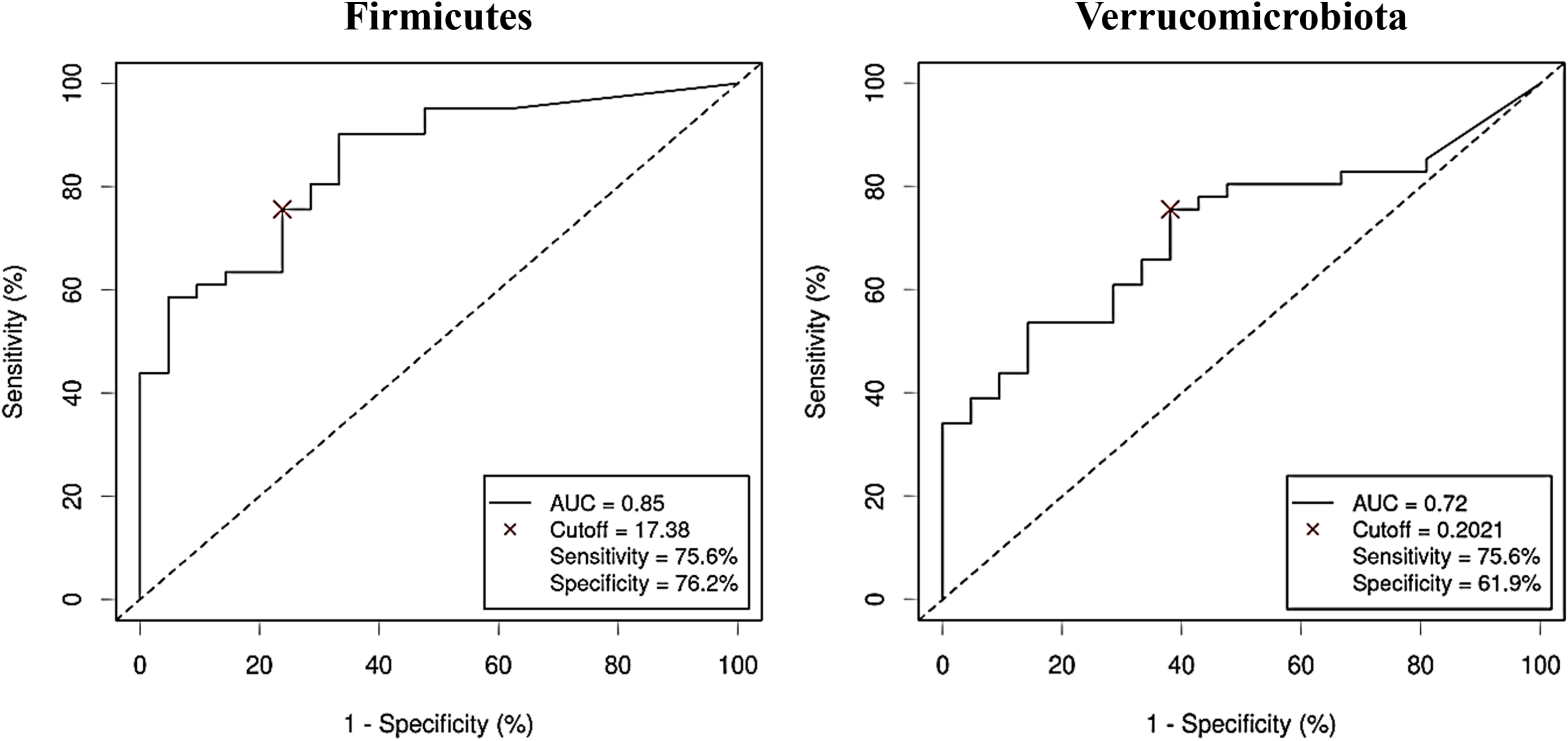
Receiver Operating Characteristic (ROC) curves showing the independent diagnostic accuracy on our study population of the relative abundance (RA) of the bacterial phyla increased in Colorectal Cancer (CRC) serum samples. AUC: area under the curve; Cutoff: RA threshold that better distinguishes between both groups, calculated with the Cutoff Finder application using the Euclidean algorithm. Event condition: CRC.

**Figure 6:**
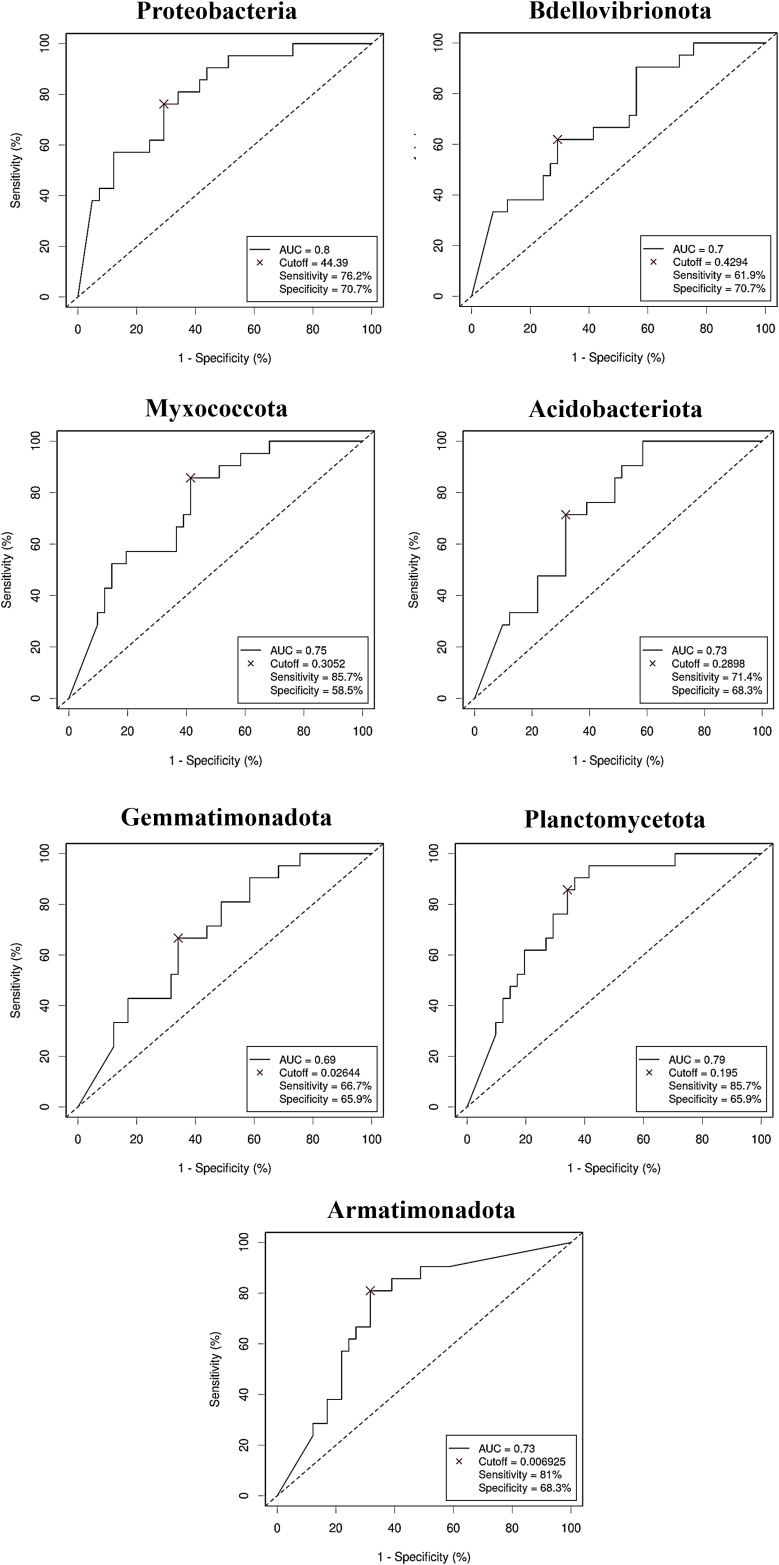
Receiver Operating Characteristic (ROC) curves showing the independent diagnostic accuracy on our study population of the relative abundance (RA) of the bacterial phyla decreased in Colorectal Cancer (CRC) serum samples. AUC: area under the curve; Cutoff: RA threshold that better distinguishes between both groups, calculated with the Cutoff Finder application using the Euclidean algorithm. Event condition: no cancer.

The diagnostic ability of the discriminative bacterial phyla between CRC and control serum samples was further confirmed by the ROC curves of their RA. Only taxa with a clear predictive effect of either CRC presence (**Figure 5**) or no cancer presence (**Figure 6**) after adjusting for age, gender and BMI were considered. The AUC was acceptable (> 0.7) for all the phyla, including Firmicutes with excellent AUC (> 0.8), except Gemmatimonadota, which reported an AUC of 0.69.

### 3.3 Serum metabolome comparison between CRC patients and controls

**Figure 7:**
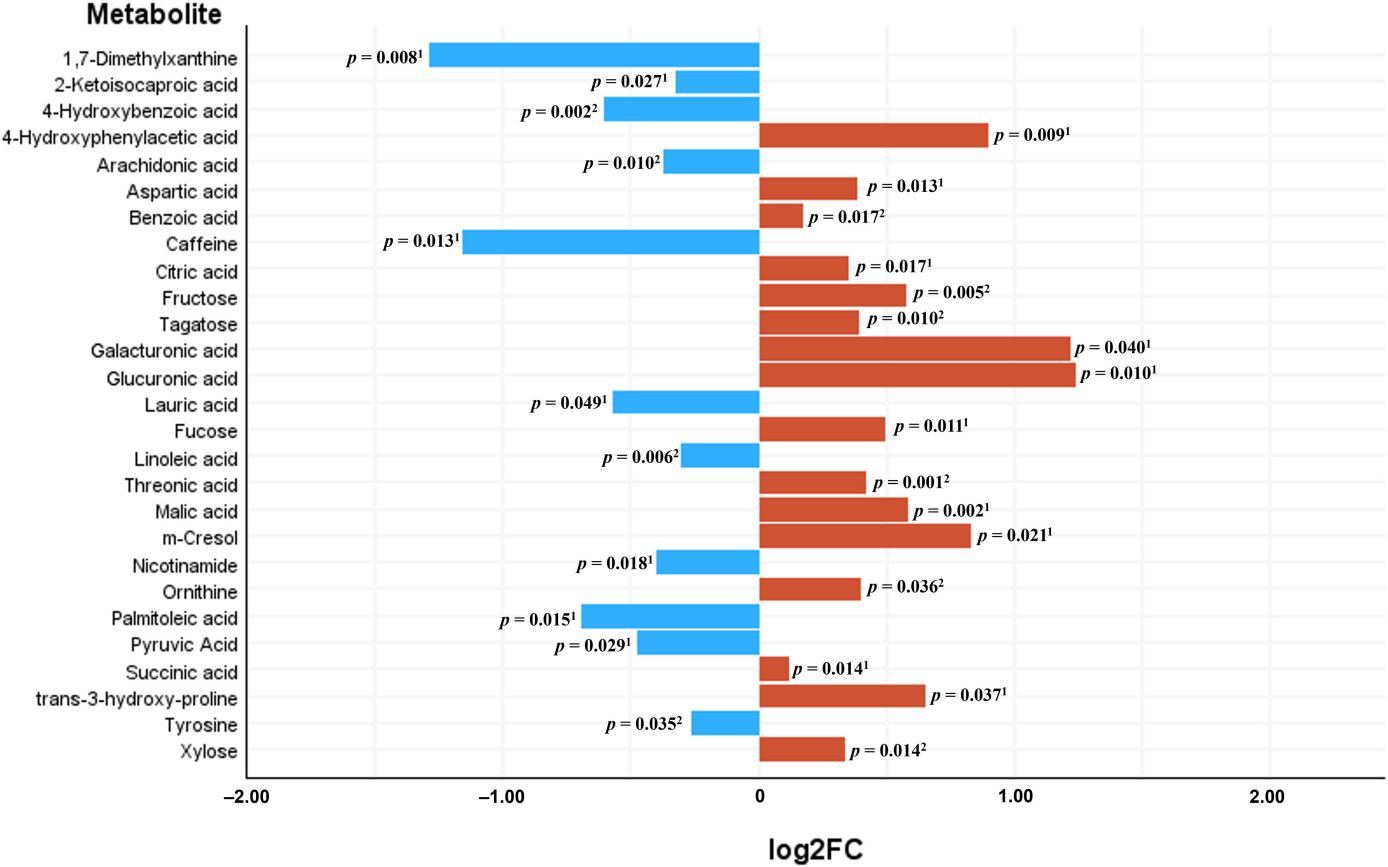
Significantly differential metabolites between sera from Colorectal Cancer (CRC) patients and sera from controls. Bars represent the base 2 logarithm of the CRC versus control fold change (FC) of the serum abundance for each metabolite. ^1^Mann-Whitney *U* test; ^2^Student’s *t* test.

Untargeted metabolomics by GC-QTOF-MS reported 121 metabolites, from which 118 were identified compounds. When the NPAs from the chromatogram of these metabolites were compared between serum from CRC patients and serum from control subjects, 27 of these metabolites were significantly different between both study groups, 16 reported as increased in CRC serum (4-hydroxyphenylacetic acid, aspartic acid, benzoic acid, citric acid, fructose, tagatose, galacturonic acid, glucuronic acid, fucose, threonic acid, malic acid, m-cresol, ornithine, succinic acid, trans-3-hydroxy-proline, xylose), and 11 as decreased (1,7-dimethylxanthine, 2-ketoisocaproic acid, 4-hydroxybenzoic acid, arachidonic acid, caffeine, lauric acid, linoleic acid, nicotinamide, palmitoleic acid, pyruvic acid and tyrosine) with respect to sera from controls. **Figure 7** represents the log2 fold change (log2FC) of abundance (NPA) of the 27 significantly increased and decreased serum metabolites in the CRC *versus* control comparison, with the P-values of the corresponding parametric or non-parametric tests.

After performing multivariate regression to adjust for age, gender and BMI value (**Table 4**), only 4 of these metabolites (4-hydroxyphenylacetic acid, threonic acid, malic acid and aspartic acid) had a significant predictive capability. Of the metabolites increased in the control group (**Table 5**) only 1,7-dimethylxanthine was significant after adjusting for confounders.

**Table 4:** Results from the logistic regression for the abundance of metabolites increased in serum samples from colorectal cancer (CRC) patients with respect to controls.

Metabolite	Unadjusted^*^ OR (95%CI)	Unadjusted^*^ *p* value	Adjusted^#^ OR (95%CI)	Adjusted^#^ *p* value
4-Hydroxyphenylacetic acid	NS (NS-1.992E+171)	0.029	NS (2.469E+78-NS)	0.042
Aspartic acid	1.226E+80 (0.016-9.413E+161)	0.055	3.218E+122 (1.036E+12-9.996E+232)	0.030
Benzoic acid	2.139E+140 (905’555.447-5.053E+274)	0.041	1.698E+169 (0-NS)	0.084
Citric acid	6.586E+78 (3.093E+8-1.402E+149)	0.028	5.145E+90 (0-1.230E+192)	0.079
Fructose	2.183E+6 (34.965-1.363E+11)	0.010	92’262.080 (0.167-5.096E+10)	0.090
Tagatose	9.012E+56 (1.969E+10-4.124E+103)	0.017	1.467E+42 (0-1.373E+99)	0.147
Galacturonic acid	NS (0-NS)	0.101	NS (0-NS)	0.106
Glucuronic acid	NS (0-NS)	0.090	NS (0-NS)	0.073
Fucose	NS (4.293E+28-NS)	0.035	2.897E+229 (0-NS)	0.360
Threonic acid	NS (1.462E+166-NS)	0.005	NS (1.460E+56-NS)	0.028
Malic acid	NS (NS-NS)	0.003	NS (NS-NS)	0.023
m-Cresol	8.588E+19 (79.541-9.271E+37)	0.030	4’097.679 (0-5.247E+23)	0.725
Ornithine	5.161E+18 (4.636-5.745E+36)	0.042	2.045E+20 (0-1.425E+44)	0.095
Succinic acid	1.234E+16 (0-1.531E+67)	0.537	1.351E+52 (0.008-2.399E+106)	0.060
Trans-3-hydroxy-proline	1.558E+11 (0-1.625E+38)	0.417	1.870E+19 (0-2.728E+53)	0.269
Xylose	1.725E+139 (1.822E+22-1.633E+256)	0.020	2.543E+80 (0-2.609E+224)	0.274

CI, confidence interval; NS, non estimable; OR, Odds ratio; ^*^Univariate regression analysis; ^#^Multivariate regression analysis adjusting for age, gender and body mass index.

**Table 5:** Results from the logistic regression for the abundance of metabolites increased in serum samples from controls with respect to colorectal cancer (CRC) patients.

Metabolite	Unadjusted^*^ OR (95%CI)	Unadjusted^*^ *p* value	Adjusted^#^ OR (95%CI)	Adjusted^#^ *p* value
1,7-Dimethylxanthine	0 (0-0)	0.043	0 (0-0.135)	0.049
2-Ketoisocaproic acid	0 (0-52.514)	0.057	0 (0-198’231.243)	0.070
4-Hydroxybenzoic acid	0 (0-0)	0.005	0 (0-7.331E+184)	0.149
Arachidonic acid	0 (0-0)	0.015	0 (0-4.239E+23)	0.248
Caffeine	0 (0-0)	0.050	0 (0-1.307E+16)	0.293
Lauric acid	0 (0-0)	0.021	3.891E+139 (0-NS)	0.552
Linoleic acid	0 (0-0)	0.012	0 (0-1.841E+86)	0.166
Nicotinamide	0 (0-0)	0.036	0 (0-NS)	0.623
Palmitoleic acid	0 (0-0)	0.009	0 (0-1.804E+131)	0.246
Pyruvic acid	0 (0-0.227)	0.034	0 (0-911’309.702)	0.381
Tyrosine	0 (0-0.396)	0.040	0 (0-1.994E+6)	0.368

CI, confidence interval; NS, non estimable; OR, Odds ratio; ^*^Univariate regression analysis; ^#^Multivariate regression analysis adjusting for age, gender and body mass index.

**Figure 8:**
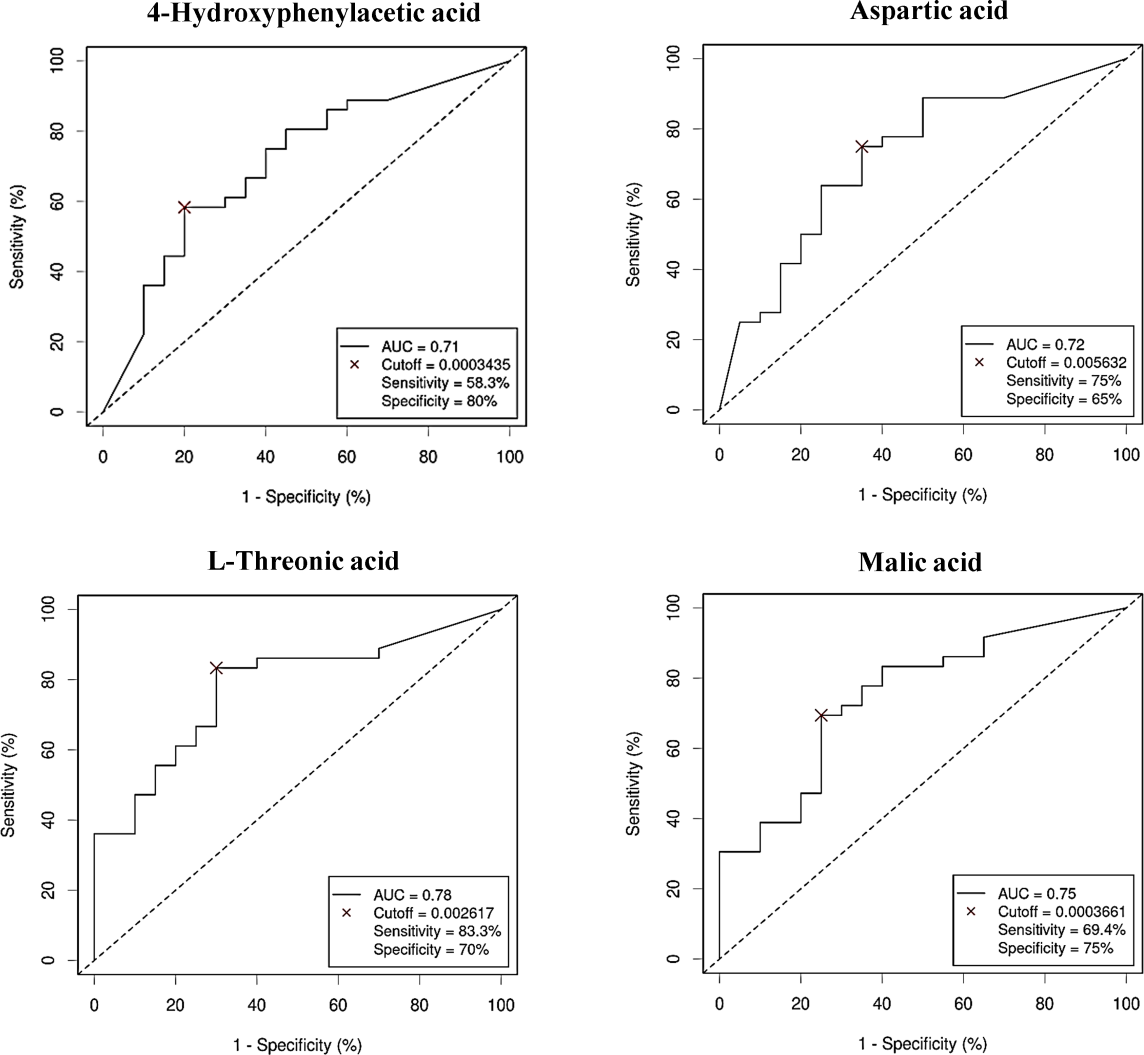
Receiver Operating Characteristic (ROC) curves showing the independent diagnostic accuracy on our study population of the abundance (normalized peak area) of the increased metabolites in Colorectal Cancer (CRC) serum samples. AUC, area under the curve; Cutoff: RA threshold that better distinguishes between both groups, calculated with the Cutoff Finder application using the Euclidean algorithm. Event condition: CRC.

**Figure 9:**
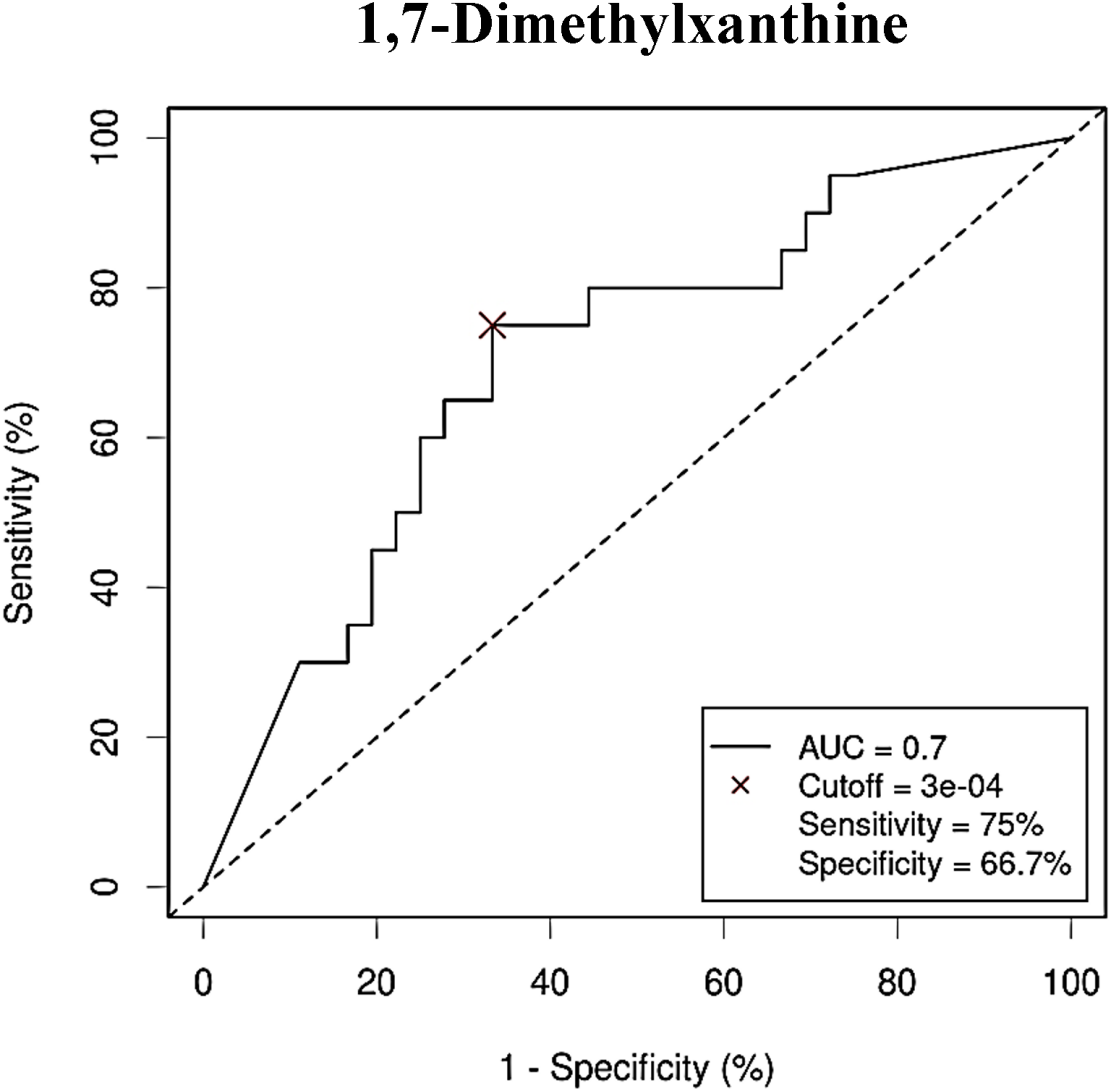
Receiver Operating Characteristic (ROC) curve showing the independent diagnostic accuracy on our study population of the abundance (normalized peak area) of 1,7-dimethylxanthine, a decreased metabolite in Colorectal Cancer (CRC) serum samples. AUC: area under the curve; Cutoff: RA threshold that better distinguishes between both groups, calculated with the Cutoff Finder application using the Euclidean algorithm. Event condition: no cancer.

As with bacterial phyla, the association between the abundances of the serum metabolites and CRC was further reported by the ROC curves, either for prediction of the CRC condition (**Figure 8**) or the no cancer condition (**Figure 9**). Again, only the five metabolites consistently incremented or diminished in CRC patients after adjusting for age, gender and BMI were considered for analysis. The optimal cut-off value for the NPA of each metabolite was determined by the Cutoff Finder application using the Euclidean algorithm. All the metabolites reported acceptable AUC values (> 0.7) when their performance was analyzed individually.

### 3.4 Integrative serum bacterial DNA-metabolome panel for CRC detection

After evaluating the capability of serum bacterial DNA and serum metabolites as predictive features for CRC by separate, both types of data were combined in a proposed diagnostic panel for CRC detection. Stepwise selection of variables was performed using both the forward selection (Wald) and backward selection (Wald) methods to select the variables that would fit better in a prediction panel.

**Figure 10:**
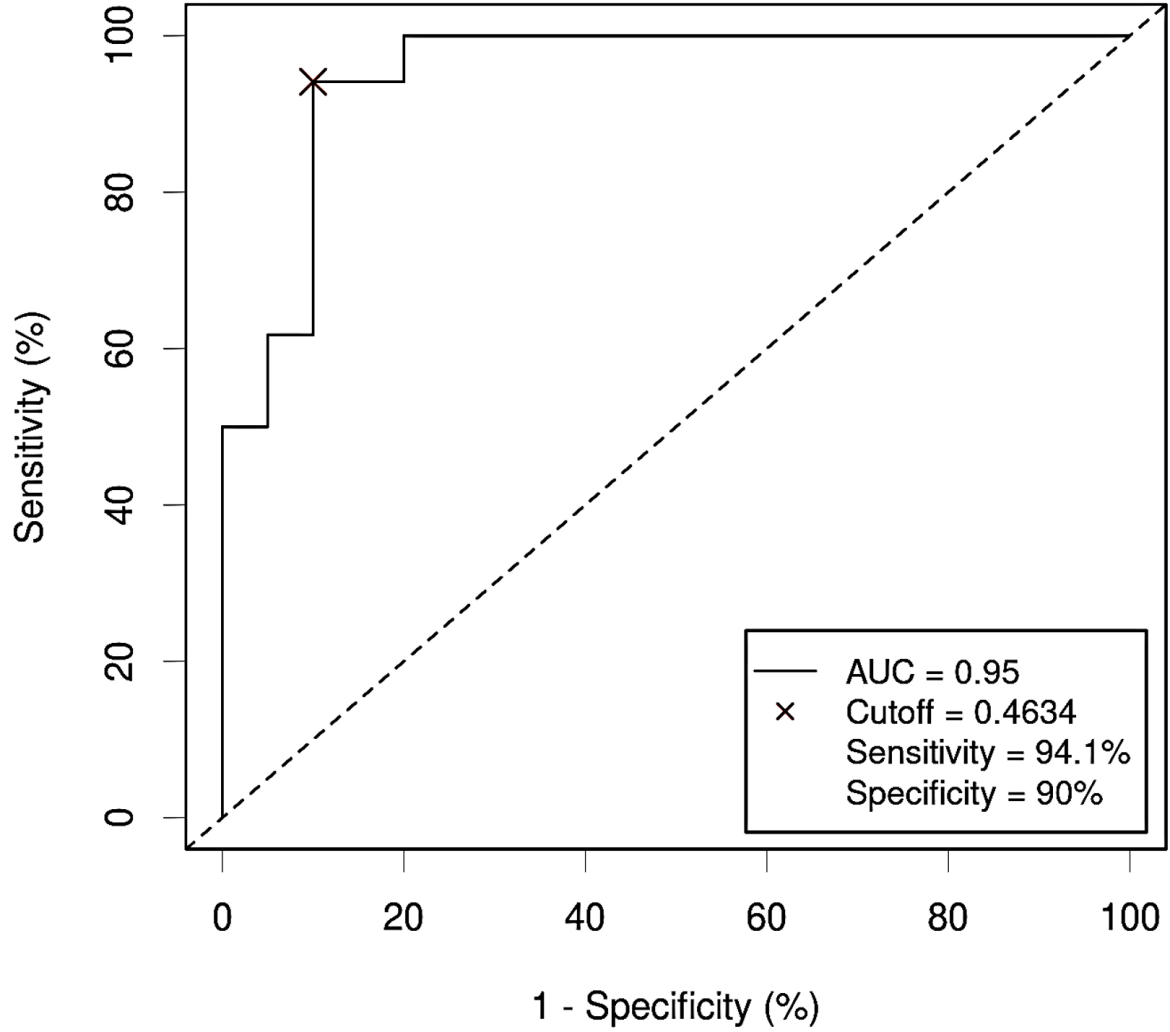
Receiver Operating Characteristic (ROC) curve showing the diagnostic accuracy for Colorectal Cancer (CRC) of the proposed panel combining relative abundance (RA) of bacterial phyla and metabolite abundance (normalized peak area) in serum samples from the study population. AUC: area under the curve; Cutoff: threshold of the probability (P) obtained by the logistic regression equation that better distinguishes both groups, calculated with the Cutoff Finder application using the Euclidean method. Event condition: CRC.

**Figure 11:**
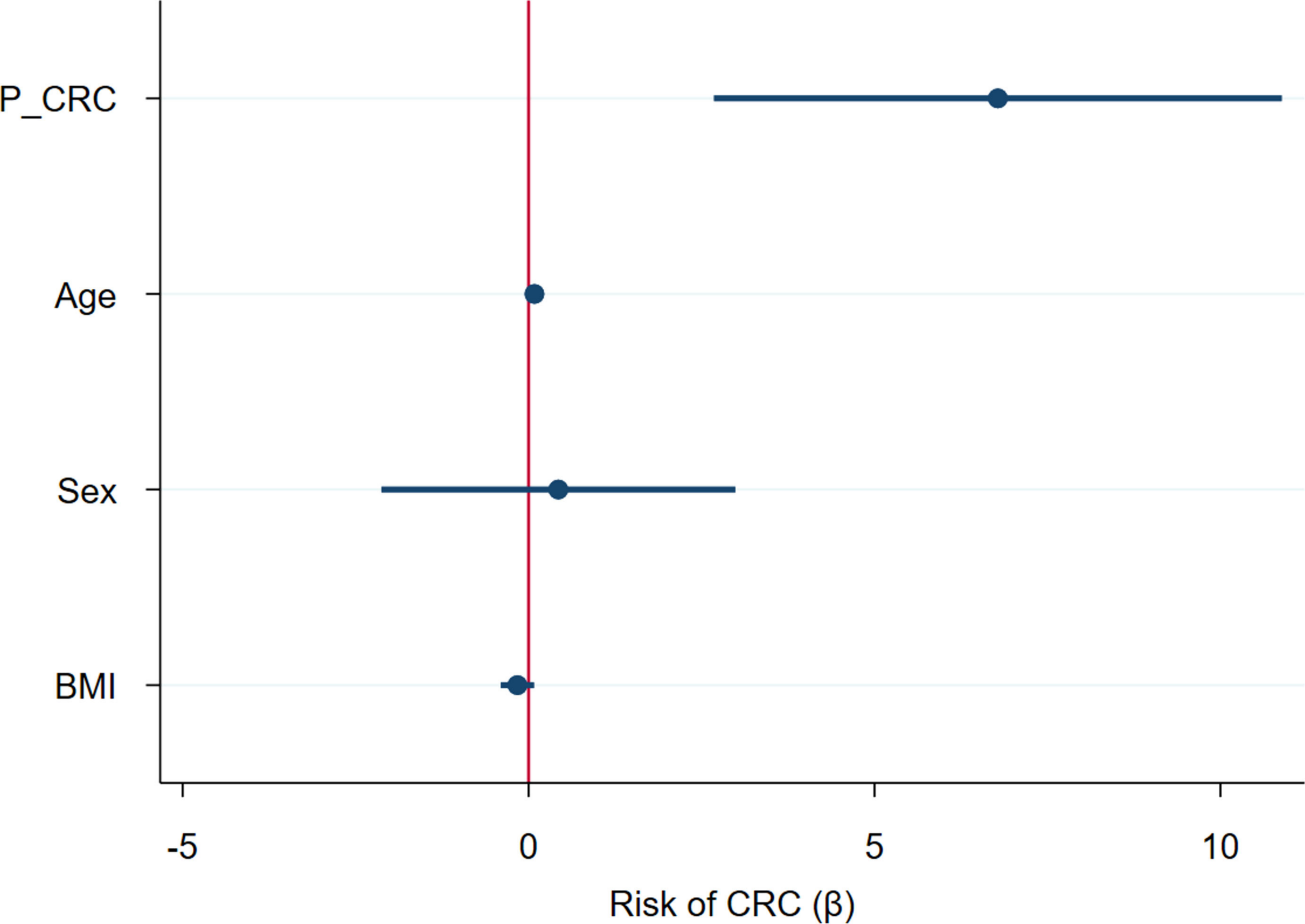
Forest plot representing the risk of Colorectal Cancer (CRC) for several variables considered together in a logistic regression model. P_CRC: CRC probability, calculated using the logistic regression equation for the proposed predictive panel. BMI: body mass index.

Input variables were bacterial phyla (RA) and metabolites (NPA) which had shown a significant association with CRC after adjusting for confounders: Firmicutes (RA), Verrucomicrobiota (RA), Proteobacteria (RA), Bdellovibrionota (RA), Acidobacteriota (RA), Planctomycetota (RA), Gemmatimonadota (RA), Armatimonadota (RA), 4-hydroxyphenylacetic acid (NPA), aspartic acid (NPA), threonic acid (NPA) and malic acid (NPA). Two variables, Myxococcota (RA) and 1,7- dimethylxanthine (NPA), were removed from the analysis as their predictive effect in the population with both metagenomic and metabolome data did not reach significance. The obtained models from both methods shared Firmicutes (RA), Planctomycetota (RA) and threonic acid (NPA) as common variables. However, in the model obtained by the forward selection method, only the variables Firmicutes (RA) and threonic acid (NPA) maintained a significant predictive effect when considered together, thus demonstrating no influence on each other’s association with CRC presence. Given these results, a final logistic regression model was obtained including only the variables Firmicutes (RA) and threonic acid (NPA) (**Table 6**). The equation obtained from this chosen model was used to calculate the probability of CRC, or P(CRC), for the study individuals, and the optimal threshold for this parameter was calculated by Cutoff Finder using the Euclidean algorithm. As shown by the ROC curve (**Figure 10**), the proposed panel had an outstanding diagnostic accuracy, with an AUC value of 0.95, a sensibility of 94.1% and a specificity of 90%. Additionally, P(CRC) was an independent risk factor for the prediction of CRC status (**Figure 11**).

**Table 6:** Logistic regression model showing the candidate serum variables in the final proposed panel for Colorectal Cancer (CRC) prediction.

Variables included	β	SE	Wald	DF	*p* value	OR (95% CI)
Firmicutes (RA)	0.249	0.081	9.569	1	0.002	1.283 (1.096-1.502)
Threonic acid (NPA)	2572.395	840.242	9.373	1	0.002	NS (NS-NS)
Constant value	-11.207	3.422	10.724	1	0.001	0 (NS-NS)

β, logistic regression coefficient; CI, confidence Interval; DF, degree of freedom; NPA, normalized peak area; NS, non estimable; OR, Odds ratio; RA, relative abundance; SE, standard error; Wald, Wald’s test parameter.

## 4 Discussion

In this study we report a putative integrative panel of serum bacterial DNA and metabolomics for the diagnosis of CRC. To our knowledge, this is the first research that merges both types of data to propose a diagnostic signature for CRC. The panel comprises two features (relative abundance of phylum Firmicutes and normalized threonic acid concentration), which enhances its attractiveness compared to other larger panels.

Metagenomic analysis did not report any relevant differences in the α-diversity indexes between the serum bacterial microbiome from the CRC and the control groups, in contrast to previously published studies. The β-diversity measures, on the contrary, were significantly different between both populations, in line with previous data (15–17). Taxonomic analysis allowed us to detect 9 bacterial phyla with differential abundance in serum samples from CRC patients with respect to those from control subjects (2 phyla increased in CRC and 7 diminished), regardless of their age, gender or BMI value. Firmicutes and Verrucomicrobiota were the two differentially increased phyla in the CRC group, and Firmicutes was the phylum selected as potential biomarker for CRC diagnosis in the variable selection process. This phylum has been reported to be increased in diverse samples from various cancer types (35) and was identified as increased in the serum from 23 metastatic and non-metastatic CRC patients with respect to their tissue samples (17). Furthermore, in our study, the ratio of Firmicutes to Bacteroidota abundance (F/B) was higher in CRC patients than in controls. The F/B ratio, which is associated with dysbiosis, constitutes an accepted relevant marker in the evaluation of the progression of metabolic diseases, including obesity, fatty liver disease and type 2 diabetes mellitus (36). Little is known about the implication of phylum Verrucomicrobiota in cancer, except for *Akkermansia muciniphila*, which plays an important role as prognostic marker in patients undergoing immunotherapy treatment. Moreover, a recent study found increased Verrucomicrobiota in the feces from gastric cancer patients (37).

Blood has been reported to contain mostly Proteobacteria (18), similarly to what we report here. According to our results, Firmicutes was the second most abundant phylum followed by Bacteroidota and Actinobacteria. In an article similar to ours, Pseudomonadota (formerly Proteobacteria) was the most prevalent phylum in serum samples (17). However, in another investigation linking changes in human serum microbiome with age and systemic inflammation, Firmicutes emerged as the dominant phylum (11). The reasons for this variability remain unclear, and it is yet to be determined whether the observed increase in Firmicutes reflects tissue-level changes.

Various mechanisms could be considered to explain the increase in Firmicutes in serum of CRC patients detected in our study. One possibility is that the inflammatory state associated with CRC (38) leads to a transient bacterial translocation (39). This inflammation would trigger a compromise of the tight junction or the uptake of microbial products by dendritic cells through the intestinal barrier. If this was confirmed, Firmicutes could also be used as serum biomarkers for other conditions such as inflammatory bowel disease. Recently, it has also been suggested that intestinal bacteria could be attracted from the intestine to the serum in search of serum nutrients, a phenomenon called bacterial vampirism (40).

Several controversies arise from blood bacterial microbiome studies. The main one is contamination, as serum constitutes a low bacterial biomass sample. To overcome this problem, some strategies need to be addressed, such as working in the best aseptic conditions and including controls from sampling to sequencing (41). In our work, samples were processed in proximal time, decreasing divergence between batches, and sample lectures were corrected by subtracting a mean signal obtained from two blanks. Moreover, the detection of 16S rRNA gene sequences in serum does not prove bacterial viability and is more likely indicative of residual microbial DNA rather than living, stable microbial communities. Thus, its detection may primarily reflect the translocation of bacterial DNA from other body locations, as previously suggested (19).

Regarding metabolomics data, we found 5 differentially distributed metabolites in the serum of CRC patients and in the control group, regardless of age, gender and BMI differences. Threonic acid remained as the metabolite that could potentially behave as diagnostic biomarker, together with the abundance of Firmicutes. This compound is a normal component of aqueous humor and blood (42). L-threonic isomer is a metabolite of ascorbic acid (vitamin C), related to ascorbate and aldarate metabolism. In addition to originating from the degradation of ascorbic acid, it also derives from threose and glycated proteins. Its circulating levels are influenced by diet and vitamin supplement intake (43).

The increase in threonic acid concentration in serum from CRC patients could derive from the threonate generated in vitamin C catabolism caused by oxidative conditions (44). Additionally, blood threonate has been previously associated with two microbial pathways involved in methanogenesis (45). Methan has been linked to colorectal carcinogenesis (46, 47), although not consistently (48). However, other authors have found decreased concentration of threonate in serum from CRC patients compared to healthy controls (49). It would be interesting to assess the contribution of diet to this variability in additional studies.

In our study we demonstrated that combining both metagenomics and metabolomics data could improve diagnostic accuracy. Thus, AUC went from 0.85 when considering isolated Firmicutes, or 0.78 for isolated threonic acid, to 0.95 for the combined panel. Many approaches can be made to combine data from different omics.

In this work, we used relative abundance for microbial metagenomics and normalized peak area for metabolomics through a logistic regression equation. Our work reinforces that the multiomics perspective can provide a more accurate status for both diagnosis and prognosis of CRC assessment (50, 51). Several genetic mutations are being used to assess patient management, but this could be completed with a comprehensive approach of other prognostic factors (52). The combination of multiomics and clinical features may provide a more thorough focus for discovering new therapeutic targets and novel alterations at the molecular level (53).

According to our results, serum Firmicutes and threonic acid were the most relevant features that could help differentiate CRC patients from the control group, with an excellent performance in this cohort (AUC = 0.95). However, our work presents several pitfalls. First, our population is modest in size, as we accounted for 64 serum samples in total, which could limit drawing firm conclusions about human microbiota variability. Secondly, significant differences in age and both gender and BMI distribution were noticeable between both study groups. These limitations were a consequence inherent to the type of population being operated on CRC and this aspect was controlled using multivariate regression techniques to mitigate the effect of potential confounders. Moreover, microbial findings in blood remain controversial, although some strategies to avoid bias were implemented, such as performing the analysis in a nearby time frame and subtracting a blank signal. Finally, considering that metabolomics could be strongly influenced by lifestyle variations in the landscape of blood extraction, metabolomic variation cannot be attributed solely to differences due to cancer occurrence. Overall, even if these results should be validated in other cohorts through multicenter studies, we consider that the findings could be relevant and applicable to the early diagnosis of colorectal cancer.

## Data Availability

The datasets presented in this study can be found in online repositories. The names of the repository/repositories and accession number(s) can be found below: http://www.ncbi.nlm.nih.gov/bioproject/1211866, BioProject ID: PRJNA1211866.
